# The impact of health worker absenteeism on patient health care seeking behavior, testing and treatment: A longitudinal analysis in Uganda

**DOI:** 10.1371/journal.pone.0256437

**Published:** 2021-08-20

**Authors:** Han Zhang, Günther Fink, Jessica Cohen

**Affiliations:** 1 Department of Global Health and Population and Harvard TH Chan School of Public Health, Boston, Massachusetts, United States of America; 2 Swiss Tropical and Public Health Institute, Basel, Switzerland; 3 Department of Epidemiology and Public Health University of Basel, Basel, Switzerland; University of Washington, UNITED STATES

## Abstract

**Introduction:**

Absenteeism of frontline health workers in public sector facilities is widespread in low-income countries. There is little quantitative evidence on how health worker absenteeism influences patient treatment seeking behavior, though low public sector utilization and heavy reliance on the informal sector are well documented in low-income settings.

**Methods:**

Using a unique panel dataset covering health facilities and households over a 10-month period in Uganda, we investigate the extent to which health worker absenteeism (defined as zero health workers present at a health facility) impacts patient care seeking behavior, testing, and treatment.

**Results:**

We find high rates of health worker absenteeism at public sector health facilities, with most of the absenteeism occurring at lower level public health clinics. On average, no health worker was present in 42% of all days monitored in lowest level public health clinics, whereas this number was less than 5% in high level public hospitals and private facilities. In our preferred empirical model with household fixed effects, we find that health worker absenteeism reduces the odds that a patient seeks care in the public sector (OR = 0.65, 95% CI = 0.44–0.95) and receives malaria testing (OR = 0.73, 95% CI = 0.53–0.99) and increases the odds of paying out-of-pocket for treatment (OR = 1.41, 95% CI = 1.10–1.80). The estimated differences in care-seeking are larger for children under-five than for the overall study population.

**Conclusions:**

The impact of health worker absenteeism on the quality of care received as well as the financial burden faced by households in sub-Saharan Africa is substantial.

## Introduction

The morbidity and mortality burden from avertible diseases such as malaria, pneumonia and HIV has declined substantially in sub-Saharan Africa over the past two decades [1]. Continued reductions in these diseases as envisioned in the Sustainable Development Goals will require a renewed effort toward high quality health systems [2]. Poor quality of care is currently estimated to account for roughly half of all amenable deaths in sub-Saharan Africa [1, 3]. High quality health systems do not only require traditional “building blocks” such as equipment, infrastructure and medical personnel, but also must be equipped to deliver care that is reliable, consistent and trusted by all people [4–6].

One cornerstone of a functioning health system is reliable access to care, meaning that health facilities are open and staffed with health workers during the expected days and hours. Empirical studies have shown very high levels of health workers not being present at facilities in low and middle-income countries (LMICs), with on average 30 to 40 percent of health facility staff being absent on days when researchers come for unannounced visits [7–12]. In the sub-Saharan African countries of Uganda, Kenya and Togo, absence rates of nearly 50% have been found [7].

High rates of health worker absenteeism could have an important impact on patient health care seeking behavior and health outcomes in LMICs, but rigorous evidence on this topic is limited. Evidence from qualitative studies suggests a potential role of health worker absenteeism in patient wait times, public sector utilization and care delays for maternal and newborn emergencies [13, 14]. Quantitative evidence of the impact of health worker absenteeism on health and health care seeking behavior is scarce. One study from Kenya found that absenteeism is associated with lower rates of prenatal HIV testing and treatment and with lower rates of facility delivery [15]. While public health workforce disruptions from strikes are of a different nature than disruptions from absenteeism, evidence suggests that strikes in LMICs have been associated with lower utilization of health care including facility-based deliveries, contraceptive use, and immunizations [16–20], with mixed effects on health outcomes [21–24]. While health worker strikes are an important form of health service interruption, strikes represent a form of acute, large-scale disruption in service delivery that may have a substantively different impact on patients than chronic health worker absenteeism.

To our knowledge, no study has explicitly examined how health worker absenteeism impacts patient care-seeking behavior, health care, or financial costs associated with acute illness in LMICs. Use of the public health care sector for common acute pediatric illnesses such as malaria, diarrhea, and pneumonia is remarkably low in sub-Saharan Africa [25–28], with some studies estimating public sector treatment seeking rates for febrile pediatric episodes below 50% [29, 30]. Use of private sector care for acute illnesses is very common, with many patients using formal private clinics or retail sector pharmacies and drug shops [25, 27, 28, 31, 32]. Studies have found that these outlets are often preferred over public facilities, because of their proximity, longer operating hours, and superior stocking of medications and equipment [33, 34]. However, the role of public health worker absenteeism in diverting patients away from the public sector and toward private and informal care—while intuitive—has not been demonstrated.

Understanding the role of public health worker absenteeism in driving care-seeking in private and informal outlets is important, because patients often receive lower quality care in these outlets than they would in the public sector. The quality of private sector care is widely variable, with many facilities staffed by unlicensed, unregulated, and untrained providers. A study from Uganda found that only 10% of febrile children treated at registered drug shops received appropriate treatment for malaria [27], and those who initially sought care at drug shops had higher odds of getting severe malaria [35]. Private sector outlets also often have limited ability to conduct diagnostic tests, have financial incentives for overtreatment, and generally high out-of-pocket (OOP) payments relative to public sector providers [31, 36].

One reason why there is limited evidence of the role of health worker absenteeism in care-seeking behavior for acute illnesses in LMICs is because of the empirical challenges in addressing this topic. Reliable routine data on staff attendance is not available in most health facilities in LMICs. While some studies have collected data by direct physical verification of provider presence through unannounced visits to health facilities [8, 10, 12, 37], this approach would be difficult and costly for the high-frequency data collection needed to create panel data. Further, most longitudinal datasets capturing patient treatment seeking behavior cannot be linked to absenteeism information at local health facilities. In this paper, we constructed absenteeism measures from health facility’s daily attendance records, which we matched to survey data on illness episodes and treatment seeking practices over a period of 10 months in six districts of Uganda. Using this unique panel dataset and a household fixed effects modeling approach, we estimate the impact of health worker absenteeism on patient health care-seeking, treatment received and OOP payments for acute illness episodes.

## Materials and methods

### Study population and context

This study was conducted in six districts in Eastern Uganda (Fig A in S1 Appendix): Budaka, Bukedea, Kibuku, Kumi, Ngora and Pallisa between July 2011 and April 2012. While under-five mortality has declined substantially in Uganda since 1980, the under-five mortality burden remains high, at 46 deaths per 1000 live births in 2018 [38]. Most of these child deaths are attributable to malaria, anemia, malnutrition, and diarrheal disease [39]. Roughly 70% of fever cases among children under-five are treated outside the home, with private sector care the sole external source for more than two thirds of these cases [40]. Health workforce shortages are common in the country, with only 0.7 skilled health professionals per 1000 people in 2019 which is far below the World Health Organization (WHO) recommended standard of 4.45 [41].

The six study districts are mostly rural and poor, with the majority of households engaged in subsistence farming. Malaria is a common cause of illness in the study districts, with 16.9% malaria prevalence among children under-five and with higher prevalence rates at the time of the study [42, 43]. Other common childhood illnesses in this region include anemia, malnutrition, diarrhea, and respiratory infections [39].

Health services in Uganda are provided through both public and private sector facilities. 55% of registered health facilities are operated by the government, 16% are private-not-for-profit, and 29% are private-for-profit [44]. Officially, health services provided through the public sector are free; however, informal payments are sometimes charged and supply chain constraints often limit facility capacity in services delivery [45, 46]. The public sector is arranged in a hierarchical system, where Health Center Levels II, III, and IV offer primary care, district and regional referral hospitals offer secondary care, and national referral hospitals and specialized institutes offer tertiary care. The private sector is composed of private clinics and hospitals, as well as retail outlets ranging from small informal, unlicensed retail drug shops and vendors to large formal pharmacies. Private sector outlets—especially retail drug shops—are widely distributed throughout Uganda, and are open for long hours and most days of the week and are easily accessible to much of the Ugandan population. However, many of them are run by attendants with no formal health or pharmacy training and are not formally registered or licensed to dispense prescription medicines.

### Data collection/survey tools

The data used in this analysis was collected as part of a cluster randomized controlled trial that took place between March 2011 and April 2012, described in further detail in Cohen et al. [47]. In the trial, drug shop vendors in selected villages were offered the opportunity to be trained in the administration of rapid diagnostic tests for malaria and then were offered access to these tests for sale at a subsidized price. The intervention had a modest effect on the stocking and selling of rapid diagnostic tests for malaria at licensed shops, with roughly 60% of intervention shops purchasing tests for sale and, among these shops, most selling only a handful of tests per month. The intervention led to a moderate, significant increase in the likelihood of febrile illnesses in the intervention communities being tested for malaria [47]. We discuss our analytic methods for accounting for the overall trial design below.

A census of all households within 92 villages across six study districts was conducted. Among these households, roughly 25 households in each village (2,366 in total) were randomly selected for study inclusion. Households were first visited for a baseline survey that included information on demographics, malaria knowledge, and health treatment seeking behavior. After the initial visit, households were re-visited every month for nine months to record information about illnesses and treatment seeking. For all surveys, the female head of household was interviewed. Respondents were asked to indicate all illnesses experienced by each household member in the past month. For every illness experienced by a household member, a detailed treatment seeking module was completed, which recorded the type and duration of illness, all treatment options used, type and costs of medication, and testing procedure.

Survey participation rates were high. Among the households selected for the baseline study, only 0.9% of households refused to participate in the study, and 5.5% were selected but either couldn’t be found or moved when the baseline survey began. Among the households who completed the baseline survey, 91.7% were still participating by month 10. The attrition was mostly from the households that moved between follow-up interviews.

All health facilities within the six study districts were visited monthly by study staff between July 2011 and April 2012. Each month facilities were given a register and asked to record the number of health workers present on each day as well as the daily stocking of certain medications and tests. The facility in-charge was provided with a small incentive to record the information. Reported facility attendance was used for the study only, and not shared with districts or other health administrative offices to avoid potential under-reporting of absenteeism. If none of the facility staff were present on the scheduled day of register collections, study staff made two additional attempts to collect the register in that month.

### Variables

#### Exposure variable

We created an absenteeism variable that was equal to one if zero health workers were present at a health facility on the day an illness started and zero otherwise. In order to construct the exposure, households first had to be assigned to a particular health facility. This is not straightforward, as many households used multiple facilities over time and many households did not use their nearest facility. Our main exposure variable considered absenteeism at a household’s “normal” facility, defined as the facility they most frequently visited over the entire study period. If a household visited two or more facilities an equal number of times during the study period, we selected the facility that was closest to the household based on geographical distance. We also conducted sensitivity analyses using two different measures of the exposure (Table A and Table B in S1 Appendix). The first considered absenteeism using the public health facility geographically closest to the household (using straight line distance). The second considered absenteeism based on the first health facility visited by the household in the previous illness episode (thus this measurement excludes every household’s first illness episode).

#### Outcome variable

We included two sets of binary outcomes in analysis. The first set is patient treatment seeking behavior, including where treatment was sought (i.e., public sector, private sector, or retail sector) and the level of health facility used to treat the illness. The second set indicates the types of treatment received and the associated short-term outcomes, including whether the patient received a malaria test, received any medications, received an antimalarial drug, received an antibiotic, paid anything out of pocket for medications, and whether the illness lasted more than one week. When a person visited multiple outlets for a particular illness episode, we recorded all of the places the person went to (up to three total) and coded outcome variables based on the care provided at all the places. For example, if a person first went to a public health facility and received a test, and then went to a private drug shop and purchased antibiotics, we coded the person as having used a public health facility and having used a drug shop and receiving both a test and antibiotics.

### Sample

A total of 134 health facilities were identified either within the six study districts based on a listing of such facilities provided by the Ministry of Health, or in nearby districts and reported as used by households in the study sample. This included 75 public facilities (71 health centers levels I-IV and 4 hospitals) and 59 private facilities (55 clinics and 4 private non-profit hospitals). 14 public facilities and 21 private facilities were dropped because they were either out of the study area, were not actually health clinics, did not report back absenteeism data, or did not consent to be monitored. This yielded a total of 99 facilities. We address how the facility sample exclusions may have impacted our results in the Discussion section.

A total of 13,994 illness episodes across 1,765 households occurring between July 2011 and April 2012 were reported. Episodes were excluded if respondents were unable to recall any facility they used (these households were included in the sensitivity analysis that considered the exposure to be absenteeism at the closest formal facility) or if they could not recall the illness start date or health worker attendance information on their illness start date was not collected. The households that experienced only one illness episode during the entire study period were also excluded as a single episode leaves no variation within households and thus cannot be used within a fixed effects analysis. This yielded a final sample of 11,595 illness episodes across 1,555 households.

### Analysis

Our primary objective was to estimate the impact of absenteeism on treatment seeking behavior and the treatment received. Our primary unit of analysis was an illness episode reported by study households. When there are multiple illness episodes for the same person across different months, we treat them as independent. Illness episodes were coded as “exposed to absenteeism” if no health worker was present at the household’s normal facility on the first day of the illness episode. As caregivers may not seek care right away but rather watch and wait, we also explored a construct of the absenteeism variable in our sensitivity analysis (Table C in S1 Appendix) where we defined absenteeism as no health workers being present in either of the *first three* days of the illness episode (and no absenteeism if health workers are present on all of the three days).

Fixed-effects logistic regression (mixed) models were used to assess the impact of absenteeism on the outcome measures. To control for seasonal effects and household characteristics, a full set of household and calendar month intercepts (fixed effects) were included in all models. The inclusion of household fixed effects means that the analysis compares treatment seeking behavior and the treatment received *within* a household during a time when the illness overlaps with an absenteeism episode vs. when the illness occurs without absenteeism. This accounts for any unobserved, confounding variables that are fixed across households. As we have multiple episodes for individuals assigned to the same facility, we clustered the robust standard errors at the facility level using Huber/White cluster robust variance estimator to account for correlation within facilities both spatially and temporally [48, 49]. We also controlled for whether or not the illness occurred on a weekend to account for the episodes that occurred on the days when a facility was not supposed to be open. We also explored alternative specifications, including a model with household random-effects, a generalized estimating equation (GEE) model and a linear regression model in the initial analysis–since results were broadly similar and the inclusion of household fixed effects minimizes the risk of confounding through household or community-specific factors, we decided to focus on a mixed model with household fixed effects as our main empirical approach.

In theory, the main intervention (drug shop training in malaria rapid diagnostic tests) could influence the relationship between absenteeism at health facilities and household health seeking behavior if, e.g., the drug shops in intervention villages were now seen as a more credible alternative to health facilities. However, any direct effect of the intervention on household treatment seeking behavior should be removed from the analysis by the household fixed effects. The only exception to this would be if the analysis included illness episodes from both before and after the introduction of the intervention. For this reason, we restrict our analysis to after the training of the drug shops.

The estimated coefficients were presented in forest plots to visualize the effect sizes and confidence intervals. We presented regression estimates both for the full sample and for the subset of illness episodes occurring among children under age five only (38.1% of illnesses), since prompt, formal treatment seeking is most important for this age group. We also conducted sensitivity analysis using two alternative measures of the exposure based on absenteeism at the closest public health facility and the health facility visited by the household in the previous illness episode. We also conducted sensitivity analyses in which the exposure was the fraction of days of the illness in which no health worker were present instead of a binary variable in Table D in S1 Appendix.

Simulations were used to determine power ex-post. Our full sample allowed us to detect a 0.19 standard deviation (SD) difference in outcome measures (i.e., care seeking behavior, treatment, and testing) with probability 0.9. In the sample of under-five children, we were able to detect a 0.31 SD difference with power 0.9.

All empirical analysis was conducted using Stata 16.0 MP statistical software package and the map in Fig A in S1 Appendix was produced using the R software package.

### Ethics statement

This study was reviewed and approved by the internal review boards of the authors’ institutes and the Uganda National Council for Science and Technology. Written consent from the female head of household was obtained at baseline for the household surveys. This included consent to be followed up with on a monthly basis and consent to speak with another caregiver in the household if the primary female caregiver was absent for an extended period of time. Verbal consent was obtained at each follow-up household survey. Permission to collect data from health facilities was obtained by District Health Officers and consent to participate in the study was collected from the facility in-charge.

### Trial registry

The trial was registered as clinical trial NCT01652365 at clinicaltrials.gov.

## Results

Our final analysis sample consists of 11,595 illness episodes across 1,555 households. Fig B in S1 Appendix shows the detailed sample selection process. Characteristics of study households at baseline and the illness episodes they experienced over the study period are presented in Table 1. Households had 6.8 members on average with 1.4 children under the age of five. 81% of households owned a mosquito net, with 3.6 people per household having slept under a bed net the night before the survey. 90% of households reported having at least one member experiencing a malaria episode, and 85% of households reported having experienced another type of illness episode, in the month preceding the baseline survey. Among children under age five, 69% had a reported malaria episode during the month before the baseline survey. Table H in S1 Appendix shows a comparison of baseline characteristics between households included in and excluded from the final analysis. In general, the included and excluded households had similar characteristics, except that the included households had more household members and were more likely to own a bicycle or mobile phone at baseline. This suggests that our final analysis sample may be slightly wealthier than the overall population in the study areas.

**Table 1:** Household characteristics, access to care, illnesses, and health care seeking.

Panel A. Households (N = 1555)	Mean (SD) / N (%)
*Household Baseline Characteristics*	
Number of household members	6.81 (3.19)
Number of children under age 5	1.43 (1.19)
Owns a mosquito net	1267 (81.5%)
Number of people who slept under a mosquito net	3.59 (2.97)
Number of people who had malaria in past 30 days	3.82 (2.96)
Number of people who had any other sickness in past 30 days	3.47 (3.07)
Number of under-five children who had malaria in past 30 days	0.99 (1.07)
Number of under-five children who had any other sickness in past 30 days	0.74 (1.04)
At least one member had malaria in past 30 days	1401 (90.1%)
At least one member had any other sickness in past 30 days	1323 (85.1%)
*Access to and use of facilities*	
Number of public facilities within a 3km radius	1.26 (1.00)
Number of private facilities within a 3km radius	1.55 (2.05)
Number of retail sectors within a 3km radius	7.38 (5.24)
Distance to closest public facility (km)	2.01 (1.73)
Distance to closest private facility (km)	3.27 (3.19)
Distance to closest retail sector (km)	0.85 (0.90)
The type of formal facility most frequently used is a:	
Private Clinic and Hospital	417 (26.8%)
Public Health Center	1014 (65.2%)
Public Hospital	124 (8.0%)
Panel B. Illnesses (N = 11595)	
Number of illness in the household per month over study period	0.75 (0.49)
Duration of this illness (days)*	5 (3–7)
Use drug shop/pharmacy to treat this illness	4610 (39.8%)
Only use drug shop/pharmacy to treat this illness	3973 (34.3%)
Use public health facility to treat this illness	3171 (27.3%)
Use private facility to treat this illness	1768 (15.2%)
Use any facility-based care to treat this illness	4538 (39.1%)
No use of external source to treat this illness at all	3084 (26.6%)
Use closest public facility to treat this illness	1833 (15.8%)
Use closest private facility to treat this illness	770 (6.6%)
Use closest formal facility to treat this illness	1819 (15.7%)
Received a malaria test for this illness	3153 (27.2%)
Took an antimalarial drug for this illness	6257 (54.0%)
Took an antibiotic for this illness	3125 (27.0%)
Took any medications for this illness	8770 (75.6%)
Number of medications taken for this illness	2.06 (1.43)
Paid OOP for medications for this illness	4168 (35.9%)
Total OOP cost for medications for this illness (USD)	1.14 (3.58)

*: median value reported, interquartile range in the parenthesis.

Fig A in S1 Appendix illustrates the spatial distribution of households and facilities. The average household had 1.3 public facilities, 1.6 private facilities and 7.4 retail drug shops within a 3 km radius (Table 1 Panel A). The average distance between a household and the nearest public facility was 2.0 km and the distance to the nearest private facility was 3.3 km. Retail sector shops were closest on average, with the average household having a retail shop within 0.9 km. Among households who sought care at a formal facility, public health centers were the most common source of treatment, used by 65.2% of households. Private clinics and hospitals were used by 26.8% of households, while public hospitals were used by 8.0%. These included households appeared to have better access to both public and private health care than the excluded households and have more retail drug shops within a 3 km radius (Table H in S1 Appendix).

Turning to the characteristics of illness episodes, the average household reported 0.8 illnesses per month, with a median illness duration of 5 days (Table 1 Panel B). Retail shops were the most common source of care, with 40% of illnesses treated in the retail sector at some point during the illness. Only 39% of illnesses were treated at a health facility (public or private), and 27% of all illnesses were treated at a public health facility. With regard to testing and treatment for the illness, 27% of illnesses were tested for malaria and 54% of illnesses were treated with an antimalarial drug. 27% of illnesses were treated with antibiotics. On average, households spent $1.14 USD OOP payments for medication.

Among the 99 facilities included in the study, 59% had at least one day with all staff absent during the study period. Overall, health workers were absent on 15% of all monitored days (Table 2). Absenteeism rates were higher at the lower level public facilities, with no health worker present on 42% of monitored days at the level II health centers and on 20% of monitored days at level III health centers. Higher level public facilities (Health Center IV and Hospitals) and private facilities (Hospitals and Clinics) had much lower rates of absenteeism (less than 5%).

**Table 2:** Health worker absenteeism by facility type.

Facility type	Fraction of days with absenteeism	Total
	Mean (SD)	
Private Clinic and Hospital	0.04 (0.09)	38
Public Health Center I	0.15 (0.14)	4
Public Health Center II	0.42 (0.48)	14
Public Health Center III	0.20 (0.31)	35
Public Health Center IV	0.04 (0.08)	5
Public Hospital	0.002 (0.002)	3
Total	0.15 (0.29)	99

Note: 1) Fraction of days with health worker absenteeism is the total number of days with zero health workers presented at a facility divided by the total number of days observed during the study period. 2) We exclude Public Health Center level I in regression analysis as they are Village Health Teams or individual Community Health Workers and are generally not considered as facility-based care.

**Fig 1:**
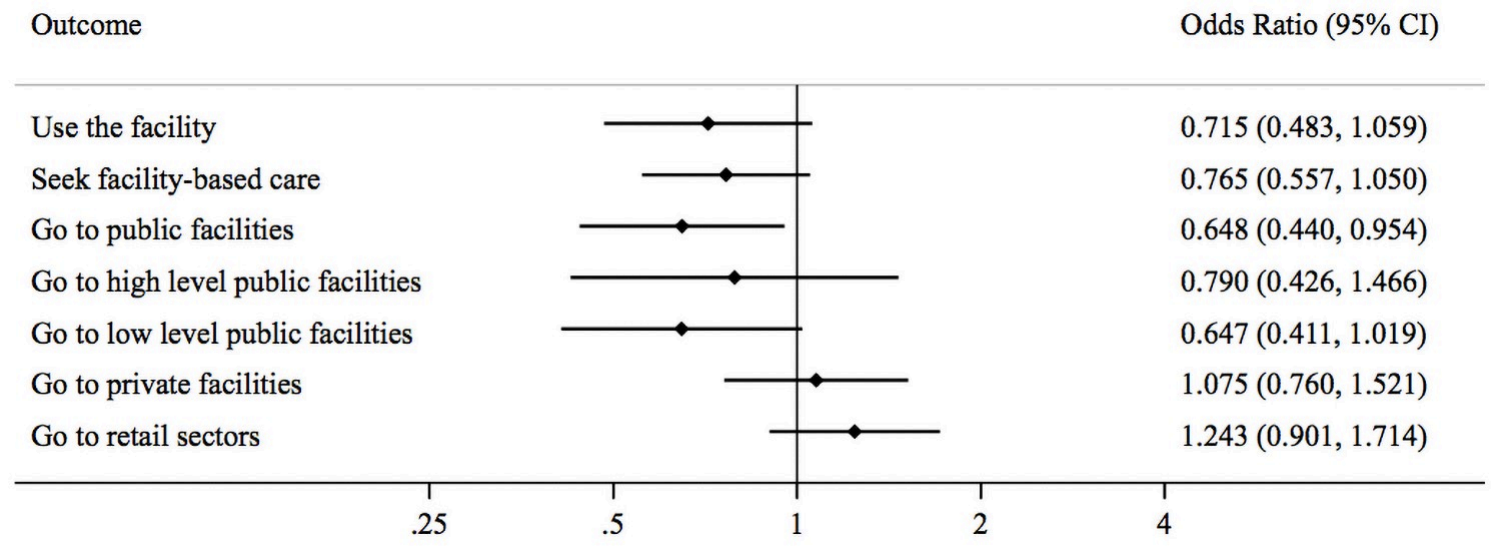
Forest plot showing the impact of health worker absenteeism on patient care seeking behavior among full sample.

Table 3 presents impact estimates both for the full sample of households (Panel A) and under-five child illnesses (Panel B). At the margin of statistical significance, absenteeism at the household’s normal health facility reduced the odds of using that facility by 28% [odds ratio (OR) = 0.72, 95% confidence interval (CI) = 0.48–1.06] and reduced the odds of receiving any facility-based care for the illness by 23% [OR = 0.77, 95% CI = 0.56–1.05] (Table 3 Panel A and Fig 1). Absenteeism led to shifts in care seeking away from lower-level public facilities, decreasing the odds of using a public facility by more than 30% [OR = 0.65, 95% CI = 0.44–0.95]. No absenteeism effects were found on the use of high-level public facilities, private facilities or retail drug shops.

**Table 3:** Impact of health worker absenteeism on patient health care seeking behavior, testing and treatment.

	Panel A: Full sample				Panel B: Under-five children			
*Health Care Seeking Behavior*	Mean	OR	p-value	95% CI	Mean	OR	p-value	95% CI
Use the facility	0.339	0.715*	0.094	[0.483, 1.059]	0.394	0.584**	0.017	[0.376, 0.907]
Seek facility-based care	0.396	0.765*	0.098	[0.557, 1.050]	0.420	0.607**	0.019	[0.400, 0.922]
Go to public facilities	0.345	0.648**	0.028	[0.440, 0.953]	0.390	0.647*	0.064	[0.409, 1.026]
Go to high level public facilities	0.293	0.790	0.455	[0.426, 1.467]	0.352	0.345*	0.087	[0.102, 1.168]
Go to low level public facilities	0.328	0.647*	0.061	[0.411, 1.020]	0.383	0.770	0.265	[0.487, 1.219]
Go to private facilities	0.297	1.075	0.681	[0.760, 1.521]	0.348	0.576*	0.088	[0.306, 1.085]
Go to retail sectors	0.455	1.243	0.185	[0.901, 1.714]	0.471	1.584**	0.039	[1.023, 2.453]
*Testing and Treatment*								
Receive malaria test	0.332	0.726**	0.041	[0.534, 0.987]	0.384	0.649*	0.100	[0.388, 1.086]
Take medications	0.677	1.085	0.638	[0.772, 1.527]	0.634	1.492*	0.064	[0.978, 2.276]
Take any antimalarial drugs	0.530	0.863	0.217	[0.684, 1.090]	0.553	1.301	0.146	[0.913, 1.854]
Take antibiotics	0.330	0.999	0.995	[0.764, 1.306]	0.364	1.118	0.513	[0.800, 1.562]
Pay OOP for medications	0.402	1.407***	0.007	[1.098, 1.802]	0.436	1.638**	0.037	[1.030, 2.604]
Illness lasts more than a week	0.267	1.029	0.846	[0.769, 1.379]	0.309	1.180	0.527	[0.708, 1.966]
*N*	*11595*				*4414*			

Note: Confidence intervals based on clustered robust standard errors at health facility level;

*** p<0.01,

** p<0.05,

* p<0.1.

**Fig 2:**
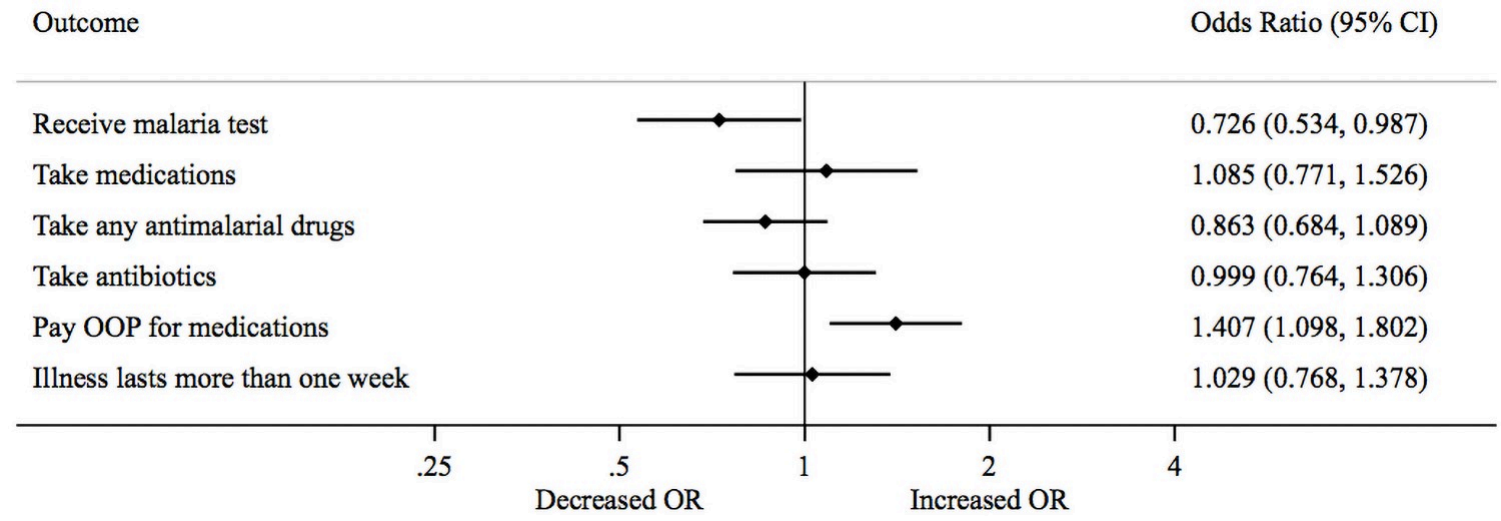
Forest plot showing the impact of health worker absenteeism on testing and treatment among full sample.

Turning to testing and treatment, absenteeism decreased the odds that an illness was tested for malaria by 27% [OR = 0.73, 95% CI = 0.53–0.99] (Table 3 Panel A and Fig 2) and increased the odds of OOP payment for medications 41% [OR = 1.41, 95% CI = 1.10–1.80]. No significant effects on the odds of taking medications or the odds that illness episode lasted more than one week were found.

**Fig 3:**
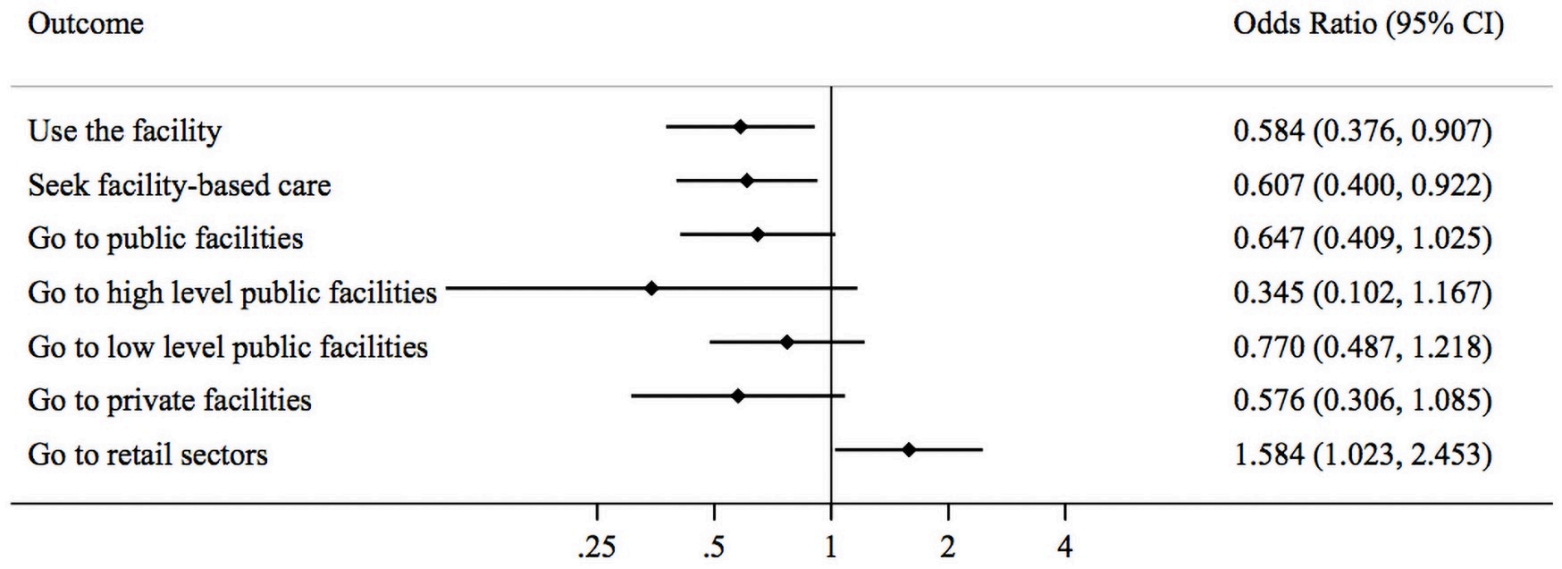
Forest plot showing the impact of health worker absenteeism on patient care seeking behavior among under-five children sample.

Among under-five children, absenteeism substantially reduced the odds of using that facility by 40% [OR = 0.58, 95% CI = 0.38–0.91] and the odds of receiving formal care by 40% [OR = 0.61, 95% CI = 0.40–0.92] (Table 3 Panel B and Fig 3). Absenteeism led to shifts in care-seeking for under-five children away from both public and private facilities toward retail drug shops, increasing the odds that the child’s illness was treated at a retail drug shop by nearly 60% [OR = 1.58, 95% CI = 1.02–2.45].

**Fig 4:**
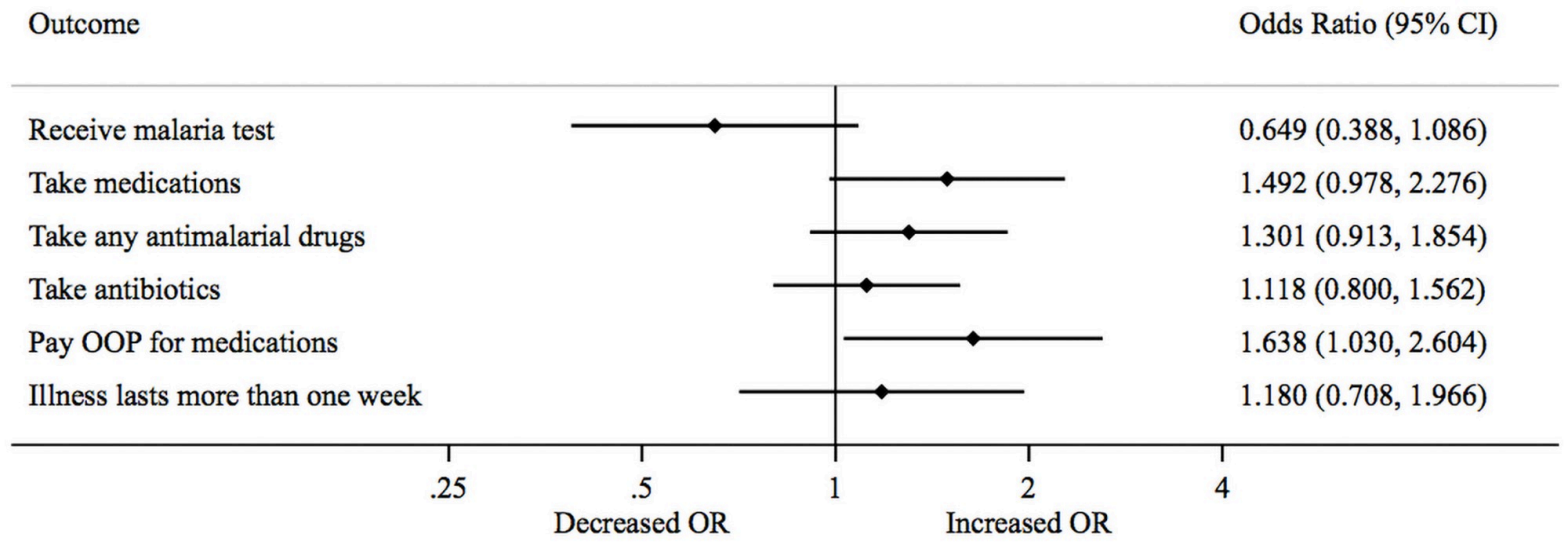
Forest plot showing the impact of health worker absenteeism on testing and treatment among under-five children sample.

Absenteeism reduced the odds of a febrile under-five child being tested for malaria by 35% [OR = 0.65, 95% CI = 0.39–1.09] and increased the odds that a child’s illness was treated with medications by nearly 50% [OR = 1.492, 95% CI = 0.98–2.28] (Table 3 Panel B and Fig 4). Like in the full sample, absenteeism substantially increased the odds that households paid out-of-pocket to treat a child’s illness by more than 60% [OR = 1.64, 95% CI = 1.03–2.60]. No absenteeism effects were found on the duration of child illness.

We conducted sensitivity analyses using two alternative measures of the exposure, the first based on absenteeism at the public facility geographically closest to the household and the second based on absenteeism at the health facility visited by the household in the previous illness episode. To account for the situation where caregivers did not seek care right away, we measured the exposure based on health workers’ presence in the first three days of the illness episodes. We also measured the exposure using the fraction of days of the illness in which no health workers were present at the facility instead of a binary variable indicating absenteeism on any day of the illness. The regression results from these sensitivity analyses appear in Tables A-D in S1 Appendix. Overall, these results are less precise but are consistent with the main analysis, suggesting that absenteeism at a formal health facility led to shifts in treatment seeking away from public facilities and toward the retail sector, declines in the odds of being tested for malaria and an increase in OOP payments.

Results for our alternative model specifications, including the linear probability model with household fixed effects, the household random-effects model, and the GEE model, are summarized in Tables E-G in S1 Appendix. Overall, the results appear stable across models. The linear probability model shows relatively narrow confidence intervals and point estimates that are consistent both directionally and in terms of the relative magnitudes with the logistic model. The direction and the magnitude of the point estimates in the random-effects and GEE specifications are similar, but confidence intervals are a bit wider and overall magnitudes a bit smaller.

## Discussion

High rates of health worker absenteeism have been found across a range of LMICs, but evidence regarding the impact of absenteeism on health care-seeking is extremely limited. The analysis presented in this study has yielded three main findings. First we find high rates of health worker absenteeism at health facilities in Uganda, with absenteeism occurring on 15% of monitored days overall and with substantially higher rates of 42% in lowest level public health clinics. Second, we find that health worker absenteeism was associated with lower health care utilization at formal public sector facilities. In particular, we find evidence of shifts away from facility-based care to drug shops and pharmacies for acute illnesses among children under-five. Third, we find that health worker absenteeism was associated with lower rates of malaria testing as well as an increased rates of OOP payments.

The high rate of health worker absenteeism we found is consistent with prior evidence on public health worker absenteeism in sub-Saharan African countries [7–9]. Our estimates of absenteeism are somewhat lower than found in previous studies. This is most likely because our measure of absenteeism—which was the fraction of days in which all staff were absent—is a stricter measure than the one used in previous studies, i.e. the fraction of health facility staff scheduled to be present on a given day who were actually present that day [8, 12, 50]. Measuring absenteeism in this way was not possible for this study, because data on the daily personnel schedule for each facility was not captured in the original data collection activities. Compared to prior measures, our approach captures the scenario when no one was at the facility at all so that the impact of individual absence cannot be attenuated by, e.g., informal task shifting to coworkers [51]. However our absenteeism measure also has some limitations which we discuss below.

Our findings on the effects of health worker absenteeism support prior qualitative findings that health worker absenteeism may cause people to delay or forego treatment seeking [13, 14]. The impact of patients switching away from facility-based care, particularly from public sector care to drug shops, is worrisome. Drug shops are widespread throughout Uganda and easy to access due to their longer opening hours [33, 52]. However, as drug shops in Uganda are often staffed by attendants with little or no formal health care training, typically do not stock diagnostic tests and sometimes sell expired or counterfeit drugs [27, 33, 53], treatment seeking at drug shops may lead to lower-quality care. Diversion of care-seeking from the public sector toward drug shops may be particularly problematic for young children because of the much higher mortality risk from common acute illnesses in this age group [54]. Given the importance of formal facility-based care for accurate diagnosis, appropriate medication prescribing, and counseling (e.g. on danger signs to look out for and when to return if the illness progresses), health worker absenteeism in our setting has far-reaching implications for the quality of care and health outcomes of the affected households and their children.

Our study suggests that public health worker absenteeism in Uganda may be leading to lower rates of diagnostic testing and higher OOP payments. The decrease in testing we found in this study is consistent with a study from Kenya demonstrating that nurse absenteeism on the day of a woman’s prenatal care visit reduced the probability of receiving HIV testing [15]. The increase in OOP payments found in this study is consistent with the qualitative literature on the impact of health worker strikes, which finds an increase in financial hardships experienced by households during service delivery disruptions in public sector care [55]. As testing is much less likely to be available in drug shops than in formal health facilities and medications are free to children in the Ugandan public sector, the decreases in testing and increases in spending associated with absenteeism in our study context could be the result of shifts in treatment seeking away from formal care and toward drug shops. However, we are not able to test formally whether this is the mechanism linking absenteeism to lower rates of testing and higher rates of spending.

While we must be cautious in extrapolating these results from Uganda to other LMICs, our study provides one of the first pieces of quantitative evidence on the behavioral, treatment, and financial ramifications of health worker absenteeism in resource-poor settings. These results could provide some indication to policy makers about potential levers for improving health service accessibility and affordability in LMICs. Our results suggest a substantially higher level of absenteeism at lower level public facilities, consistent with previous findings from India [12]. This highlights the importance of designing interventions that specifically target lower level public facilities in addressing absenteeism in LMICs. Evidence-based interventions addressing absenteeism or improving health worker performance in low-level public sector facilities in Uganda and similar settings is sparse [56, 57]. A range of innovative solutions to health worker absenteeism, from financial incentives to technological solutions such as biometric monitoring systems, have been tested in low-income settings [37, 58–60]. Yet in many cases the effects, if any, appear to be temporary, and difficult to sustain when the political environment is not conducive to change [37, 58–60]. Effective interventions that incorporate the broader political and health system context are needed to address chronic absenteeism among public health sector employees.

Our results should be interpreted in light of several limitations. First, our absenteeism measures are relatively coarse and may suffer from measurement error and reporting bias. For example, the facility in-charge (who filled in the absenteeism records) may have forgotten to record the data each day and/or may have been absent him or herself on certain days, which could lead to measurement error in our absenteeism variable. Although we told the health facility in-charges that the data would be kept confidential and would not be reported to government or any external groups, it is still possible that in-charges under-reported absenteeism because of fears or sanctioning or social desirability bias. This would cause us to under-estimate absenteeism rates but may not introduce bias in the estimates of the association between absenteeism and the studied outcomes. Our measure of absenteeism was also limited in that it only captured full-day absenteeism, but not late arrival and early exit, which may be an even more common form of service interruption in this setting. We also do not have information on the cadres or types of absent worker (e.g., doctors or nurses, clinical staff or administrative staff), which may influence the care received by patients. Finally, our measure of the impact of absenteeism in the main analysis only captured short-term behavioral responses to absenteeism, and did not capture any long-term responses or coping strategies responding to consistent absenteeism or facility closure.

Our results should also be interpreted in light of several sample exclusions. First, we are missing data from 35 facilities (11 were enrolled in the study but didn’t report absenteeism data and 24 were not enrolled at all, either because they were outside of the study area, were not actually health clinics, refused participation or other reasons). If some of these facilities did not report data or did not participate because of fear of sharing absenteeism data that could influence the interpretation and generalizability of our results. Further, some households had to be excluded from the sample either because they never used a facility or could not recall which facility they used. Our analysis suggests that these excluded households were slightly less wealthy and had somewhat worse access to care as compared to included households. Because our analysis used logistic fixed effects regression, the analysis dropped households with no variation in the main outcome variables (roughly 15–20% of observations) so our results should also be interpreted with that in mind. Finally, our study sample included only villages that had at least one licensed drug shop. While not overly restrictive since drug shops are widespread in Uganda, the sample may be less representative of the most rural or remote locations.

Finally, our study had several limitations related to outcomes measured in our dataset. Namely, we did not directly capture failed attempts at care seeking due to health worker absenteeism, so we use a household’s typically (during the entire study period) used facility as a proxy for the facility people intended to go. This measure could underestimate the impact of absenteeism on treatment seeking if people adjust their expectations and behavior over time in response to chronic absenteeism. This concern are somewhat ameliorated by the robustness of the results to different measures of the exposure (i.e., the measure using the health facility visited in the previous illness episode). Further, the data available did not allow us to assess whether absenteeism was associated with delays in care seeking or with ultimate illness resolution and health outcomes. We were able to evaluate the impact of absenteeism on the length of illness and found no significant impacts. Last, we analyzed data collected within a study focusing on the provision of rapid tests for malaria through drug shops. While the availability of drug shops did not change during periods with and without absenteeism, it is possible that differential responses could have been observed in other settings.

Overall, we find that the notably high rates of health worker absenteeism in this region of Uganda, particularly in low level public sector health facilities, are associated with reductions in formal care seeking and with increased use of retail sector drug shops. Chronic absenteeism of frontline health workers could shift health care seeking away from formal facilities to drug shops and pharmacies, reduce the likelihood of receiving diagnostic tests, and increase the financial burden to households. Future research should address the potential role that chronically high rates of health worker absenteeism may be having on population health outcomes. These findings underscore the importance of designing effective interventions to address health workforce absenteeism in public sector as a central component of health system strengthening in LMICs.

## Supporting information

S1 Appendix Figures and tables. (DOCX)

S2 Appendix Household follow-up survey instrument. (DOC)

S3 Appendix Facility survey instrument. (DOC)
