# Supplementary material for: The impact of health worker absenteeism on patient health care seeking behavior, testing and treatment: A longitudinal analysis in Uganda
Source: PLoS One. 2021 Aug 20;16(8):e0256437. doi: 10.1371/journal.pone.0256437 (PMC8378719; doi:10.1371/journal.pone.0256437)
Supplement: S1 Appendix — (DOCX) [file pone.0256437.s001.docx]

**Fig A. Distribution of households, health Facilities, and drug shops in study area**


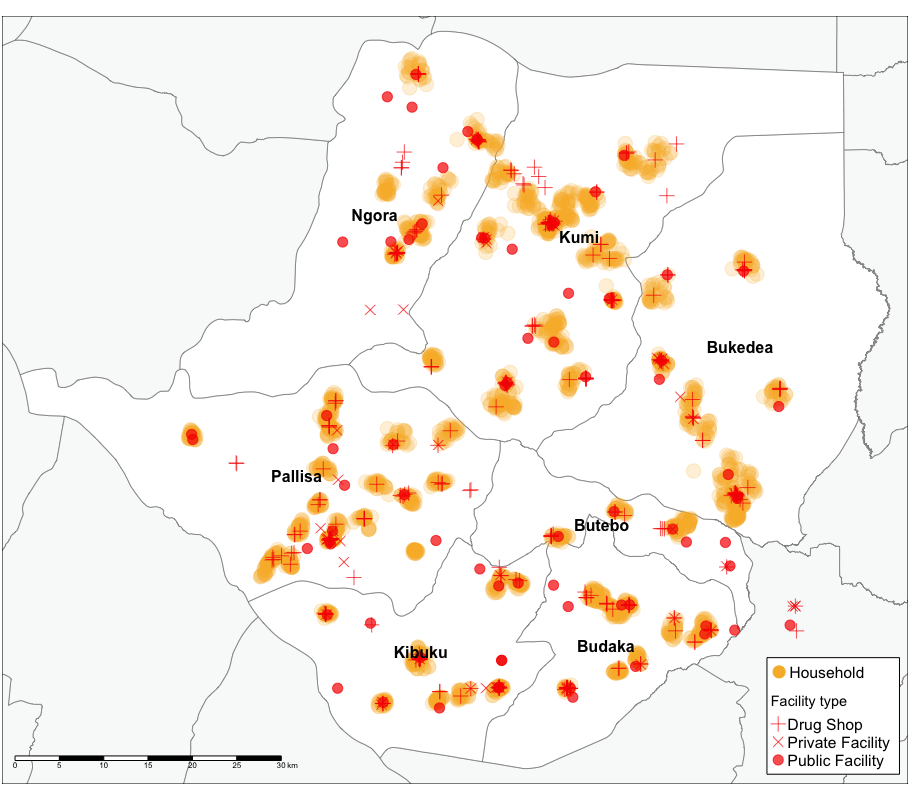


Note: 1) The figure displays the geographic distribution of households and health facilities included for data collection; 2) Butebo was a sub-county in Pallisa District during the study period, and became a district since July 2017 following requests for district status by local residents. 3) Data source for the background map is from the Humanitarian Data Exchange from the United Nations Office for the Coordination of Humanitarian Affairs (OCHA), <https://data.humdata.org/dataset/uganda-administrative-boundaries-admin-1-admin-3>

**Fig B. Process of sample restriction**

Non-eligible cases:

-  Illnesses not occurred in facility monitoring period (case n = 10660)

Excluding households with incomplete data:

-  Households cannot recall the name of the facility they used (household n = 90)

Eligible sample

(household n = 1765, case n = 13994)

Total households and cases surveyed

(household n = 2366, case n = 30613)

Excluding cases with incomplete data:

-  The start date of illness episode cannot be recalled (case n = 787)

-  Illness occurred when facility records didn’t record absent information (case n = 716)

Analysis sample

(household n = 1555, case n = 11595)

Non-eligible households:

-  Households never used health facilities (household n = 318)

-  Households whose matched facility has consecutive staff absence for more than 3 weeks (household n = 329)

Excluding households not suitable for fixed effect analysis:

-  Households only experienced one illness episode (household n = 109)

Note: a total of 13,994 illness episodes across 1,765 households occurring between July 2011 and April 2012 was collected for the analysis. A further 90 households (864 illnesses) were excluded because they cannot recall the name of the facility they were using (these households are included in the sensitivity analysis that considers the exposure to be absenteeism at the closest formal facility). 787 illness episodes across 412 households were dropped because the start date of those episodes cannot be recalled. A further 716 illnesses across 398 households were dropped because they occurred at a date when the facility records didn’t record whether their staff was absent. 109 households (109 illnesses) were dropped because those households experienced only one illness episode during the entire study period, which leaves no variation in treatment seeking behavior within households and thus cannot be used within a fixed effects analysis. This leaves a final analysis sample of 11,595 illness episodes across 1,555 households.

**Table A. Impact of health worker absenteeism at the geographically closest public health facility**

|  | A. Full sample | | | |  | B. Under-five children | | | |
| --- | --- | --- | --- | --- | --- | --- | --- | --- | --- |
| *Health Care Seeking Behavior* | Mean | OR | p-value | 95% CI |  | Mean | OR | p-value | 95% CI |
| Use the facility | 0.327 | 0.606* | 0.090 | [0.340, 1.082] |  | 0.406 | 0.593** | 0.037 | [0.363, 0.968] |
| Seek facility-based care | 0.381 | 0.844 | 0.234 | [0.638, 1.116] |  | 0.425 | 0.779 | 0.159 | [0.550, 1.103] |
| Go to public facilities | 0.351 | 0.725* | 0.058 | [0.521, 1.010] |  | 0.402 | 0.731 | 0.145 | [0.480, 1.114] |
| Go to high level public facilities | 0.285 | 0.751 | 0.316 | [0.430, 1.314] |  | 0.350 | 0.525 | 0.248 | [0.176, 1.567] |
| Go to low level public facilities | 0.344 | 0.796 | 0.262 | [0.534, 1.186] |  | 0.401 | 0.800 | 0.273 | [0.536, 1.193] |
| Go to private facilities | 0.290 | 1.149 | 0.375 | [0.845, 1.564] |  | 0.362 | 0.806 | 0.492 | [0.435, 1.492] |
| Go to retail sectors | 0.451 | 1.122 | 0.488 | [0.810, 1.554] |  | 0.467 | 1.426* | 0.054 | [0.993, 2.046] |
| *Testing and Treatment* |  |  |  |  |  |  |  |  |  |
| Receive malaria test | 0.304 | 0.830 | 0.208 | [0.622, 1.109] |  | 0.367 | 0.814 | 0.444 | [0.479, 1.380] |
| Take medications | 0.668 | 0.944 | 0.676 | [0.721, 1.236] |  | 0.620 | 1.248 | 0.115 | [0.948, 1.643] |
| Take any antimalarial drugs | 0.510 | 0.764** | 0.017 | [0.613, 0.952] |  | 0.542 | 0.997 | 0.982 | [0.745, 1.334] |
| Take antibiotics | 0.323 | 1.083 | 0.516 | [0.852, 1.377] |  | 0.363 | 1.182 | 0.395 | [0.804, 1.737] |
| Pay OOP for medications | 0.405 | 1.348*** | 0.005 | [1.093, 1.662] |  | 0.436 | 1.703*** | 0.001 | [1.233, 2.353] |
| Illness lasts more than a week | 0.263 | 1.200 | 0.278 | [0.863, 1.668] |  | 0.305 | 0.963 | 0.865 | [0.626, 1.482] |
| *N* | *10778* | | | |  | *4067* | | | |

Note: 1) Absenteeism is measured as zero health workers were present at the public health facility geographically closest to the household on the day an illness started. 2) Confidence intervals based on cluster-robust standard errors grouped at the health facility level.; *** p<0.01, ** p<0.05, * p<0.1.

**Table B. Impact of health worker absenteeism at the health facility used by households in previous illness episode**

|  | A. Full sample | | | |  | B. Under-five children | | | |
| --- | --- | --- | --- | --- | --- | --- | --- | --- | --- |
| *Health Care Seeking Behavior* | Mean | OR | p-value | 95% CI |  | Mean | OR | p-value | 95% CI |
| Use the facility | 0.361 | 0.678 | 0.112 | [0.420, 1.096] |  | 0.412 | 0.622 | 0.105 | [0.351, 1.103] |
| Seek facility-based care | 0.418 | 0.790 | 0.260 | [0.525, 1.190] |  | 0.441 | 0.709 | 0.276 | [0.382, 1.316] |
| Go to public facilities | 0.365 | 0.616** | 0.034 | [0.393, 0.965] |  | 0.409 | 0.632* | 0.085 | [0.375, 1.066] |
| Go to high level public facilities | 0.313 | 0.787 | 0.560 | [0.352, 1.761] |  | 0.360 | 0.218 | 0.150 | [0.027, 1.735] |
| Go to low level public facilities | 0.347 | 0.623* | 0.097 | [0.356, 1.090] |  | 0.405 | 0.784 | 0.410 | [0.439, 1.399] |
| Go to private facilities | 0.310 | 1.224 | 0.283 | [0.846, 1.772] |  | 0.357 | 0.920 | 0.845 | [0.401, 2.114] |
| Go to retail sectors | 0.444 | 1.337 | 0.107 | [0.939, 1.904] |  | 0.463 | 1.541 | 0.103 | [0.916, 2.592] |
| *Testing and Treatment* |  |  |  |  |  |  |  |  |  |
| Receive malaria test | 0.349 | 0.787 | 0.191 | [0.550, 1.127] |  | 0.398 | 0.684 | 0.195 | [0.385, 1.215] |
| Take medications | 0.673 | 1.150 | 0.530 | [0.743, 1.782] |  | 0.632 | 1.324 | 0.321 | [0.761, 2.305] |
| Take any antimalarial drugs | 0.535 | 0.925 | 0.541 | [0.719, 1.189] |  | 0.561 | 1.300 | 0.285 | [0.803, 2.103] |
| Take antibiotics | 0.330 | 1.012 | 0.942 | [0.733, 1.396] |  | 0.368 | 1.030 | 0.887 | [0.684, 1.551] |
| Pay OOP for medications | 0.390 | 1.716*** | 0.000 | [1.359, 2.168] |  | 0.428 | 2.056*** | 0.001 | [1.352, 3.126] |
| Illness lasts more than a week | 0.268 | 0.972 | 0.858 | [0.716, 1.321] |  | 0.309 | 0.955 | 0.858 | [0.580, 1.574] |
| *N* | *10205* | | | |  | *3957* | | | |

Note: 1) Absenteeism is measured as zero health workers were present at the first health facility visited by the household in the previous illness episode. 2) Confidence intervals based on cluster- robust standard errors grouped at the health facility level.; *** p<0.01, ** p<0.05, * p<0.1.

**Table C. Impact of health worker absenteeism using the first three days measure**

|  | A. Full sample | | | |  | B. Under-five children | | | |
| --- | --- | --- | --- | --- | --- | --- | --- | --- | --- |
| *Health Care Seeking Behavior* | Mean | OR | p-value | 95% CI |  | Mean | OR | p-value | 95% CI |
| Use the facility | 0.339 | 0.828 | 0.238 | [0.605, 1.133] |  | 0.394 | 0.827 | 0.430 | [0.516, 1.326] |
| Seek facility-based care | 0.396 | 0.728** | 0.026 | [0.550, 0.963] |  | 0.420 | 0.728 | 0.131 | [0.482, 1.099] |
| Go to public facilities | 0.345 | 0.743* | 0.062 | [0.544, 1.015] |  | 0.390 | 1.005 | 0.984 | [0.649, 1.555] |
| Go to high level public facilities | 0.293 | 0.856 | 0.633 | [0.452, 1.622] |  | 0.352 | 0.612 | 0.251 | [0.265, 1.415] |
| Go to low level public facilities | 0.328 | 0.758 | 0.110 | [0.539, 1.064] |  | 0.383 | 1.274 | 0.250 | [0.843, 1.924] |
| Go to private facilities | 0.297 | 0.829 | 0.204 | [0.621, 1.107] |  | 0.348 | 0.539** | 0.014 | [0.329, 0.883] |
| Go to retail sectors | 0.455 | 1.492*** | 0.003 | [1.148, 1.938] |  | 0.471 | 1.619*** | 0.004 | [1.167, 2.246] |
| *Testing and Treatment* |  |  |  |  |  |  |  |  |  |
| Receive malaria test | 0.332 | 0.767** | 0.048 | [0.589, 0.998] |  | 0.384 | 0.749 | 0.146 | [0.507, 1.106] |
| Take medications | 0.677 | 1.265* | 0.091 | [0.964, 1.662] |  | 0.634 | 1.513*** | 0.006 | [1.126, 2.031] |
| Take any antimalarial drugs | 0.530 | 1.025 | 0.840 | [0.806, 1.303] |  | 0.553 | 1.365** | 0.033 | [1.025, 1.819] |
| Take antibiotics | 0.330 | 1.099 | 0.391 | [0.886, 1.362] |  | 0.364 | 1.292 | 0.154 | [0.909, 1.837] |
| Pay OOP for medications | 0.402 | 1.439*** | 0.000 | [1.184, 1.750] |  | 0.436 | 1.486** | 0.022 | [1.058, 2.087] |
| Illness lasts more than a week | 0.267 | 0.918 | 0.402 | [0.752, 1.121] |  | 0.309 | 1.021 | 0.894 | [0.753, 1.383] |
| *N* | *11595* | | | |  | *4414* | | | |

Note: 1) Absenteeism is defined as no health workers being present in either of the first three days of the illness episode at the household’s normal facility (and no absenteeism if at least one health worker is present on all of the three days). 2) Confidence intervals based on cluster-robust standard errors grouped at the health facility level; *** p<0.01, ** p<0.05, * p<0.1.

**Table D. Impact of health worker absenteeism using the fraction of days measure**

|  | A. Full sample | | | |  | B. Under-five children | | | |
| --- | --- | --- | --- | --- | --- | --- | --- | --- | --- |
| *Health Care Seeking Behavior* | Mean | OR | p-value | 95% CI |  | Mean | OR | p-value | 95% CI |
| Use the facility | 0.339 | 0.879 | 0.563 | [0.568, 1.360] |  | 0.394 | 0.632* | 0.0804 | [0.378, 1.057] |
| Seek facility-based care | 0.396 | 0.667* | 0.065 | [0.434, 1.026] |  | 0.420 | 0.504** | 0.0191 | [0.284, 0.894] |
| Go to public facilities | 0.345 | 0.657* | 0.065 | [0.421, 1.026] |  | 0.390 | 0.743 | 0.3239 | [0.412, 1.341] |
| Go to high level public facilities | 0.293 | 0.799 | 0.616 | [0.333, 1.919] |  | 0.352 | 0.765 | 0.7246 | [0.173, 3.393] |
| Go to low level public facilities | 0.328 | 0.680 | 0.159 | [0.397, 1.164] |  | 0.383 | 0.767 | 0.3398 | [0.445, 1.322] |
| Go to private facilities | 0.297 | 1.000 | 0.999 | [0.631, 1.586] |  | 0.348 | 0.358** | 0.0209 | [0.150, 0.856] |
| Go to retail sectors | 0.455 | 1.382 | 0.176 | [0.865, 2.210] |  | 0.471 | 2.588** | 0.0167 | [1.188, 5.639] |
| *Testing and Treatment* |  |  |  |  |  |  |  |  |  |
| Receive malaria test | 0.332 | 0.717 | 0.228 | [0.417, 1.233] |  | 0.384 | 0.617 | 0.130 | [0.330, 1.153] |
| Take medications | 0.677 | 1.149 | 0.647 | [0.633, 2.086] |  | 0.634 | 1.786 | 0.126 | [0.850, 3.754] |
| Take any antimalarial drugs | 0.53 | 0.836 | 0.374 | [0.564, 1.240] |  | 0.553 | 1.570* | 0.097 | [0.922, 2.674] |
| Take antibiotics | 0.33 | 1.119 | 0.657 | [0.680, 1.842] |  | 0.364 | 1.130 | 0.728 | [0.568, 2.247] |
| Pay OOP for medications | 0.402 | 1.855*** | 0.001 | [1.305, 2.638] |  | 0.436 | 2.550*** | 0.005 | [1.338, 4.863] |
| Illness lasts more than a week | 0.267 | 1.388 | 0.114 | [0.924, 2.085] |  | 0.309 | 1.279 | 0.517 | [0.607, 2.697] |
| *N* | *11595* | | | |  | *4414* | | | |

Note: 1) Absenteeism is measured as the fraction of the illness in which no health worker were present at the household’s normal facility. 2) Confidence intervals based on cluster-robust standard errors grouped at the health facility level; *** p<0.01, ** p<0.05, * p<0.1.

**Table E. Alternative model: linear probability model with household fixed effects**

|  | A. Full sample | | | |  | B. Under-five children | | | |
| --- | --- | --- | --- | --- | --- | --- | --- | --- | --- |
| *Health Care Seeking Behavior* | Mean | Coef. | p-value | 95% CI |  | Mean | Coef. | p-value | 95% CI |
| Use the facility | 0.298 | -0.049* | 0.089 | [-0.105, 0.007] |  | 0.302 | -0.069*** | 0.009 | [-0.120, -0.018] |
| Seek facility-based care | 0.391 | -0.045* | 0.088 | [-0.097, 0.006] |  | 0.388 | -0.077** | 0.013 | [-0.137, -0.018] |
| Go to public facilities | 0.273 | -0.062** | 0.020 | [-0.113, -0.011] |  | 0.270 | -0.059** | 0.041 | [-0.115, -0.003] |
| Go to high level public facilities | 0.092 | -0.008 | 0.451 | [-0.030, 0.013] |  | 0.083 | -0.029** | 0.023 | [-0.053, -0.005] |
| Go to low level public facilities | 0.168 | -0.053** | 0.029 | [-0.099, -0.006] |  | 0.176 | -0.032 | 0.170 | [-0.076, 0.013] |
| Go to private facilities | 0.152 | 0.005 | 0.755 | [-0.026, 0.035] |  | 0.154 | -0.045** | 0.039 | [-0.087, -0.003] |
| Go to retail sectors | 0.398 | 0.038 | 0.179 | [-0.017, 0.094] |  | 0.397 | 0.082** | 0.031 | [0.009, 0.156] |
| *Testing and Treatment* |  |  |  |  |  |  |  |  |  |
| Receive malaria test | 0.272 | -0.044** | 0.047 | [-0.087, -0.001] |  | 0.289 | -0.063* | 0.093 | [-0.135, 0.010] |
| Take medications | 0.756 | 0.012 | 0.589 | [-0.032, 0.056] |  | 0.748 | 0.053** | 0.046 | [0.002, 0.105] |
| Take any antimalarial drugs | 0.540 | -0.026 | 0.240 | [-0.070, 0.017] |  | 0.589 | 0.043 | 0.128 | [-0.012, 0.098] |
| Take antibiotics | 0.270 | -0.001 | 0.973 | [-0.048, 0.047] |  | 0.277 | 0.021 | 0.527 | [-0.043, 0.085] |
| Pay OOP for medications | 0.359 | 0.062*** | 0.009 | [0.017, 0.107] |  | 0.374 | 0.083* | 0.062 | [-0.003, 0.168] |
| Illness lasts more than a week | 0.209 | 0.002 | 0.941 | [-0.038, 0.041] |  | 0.195 | 0.019 | 0.540 | [-0.042, 0.080] |
| *N* | *11595* | | | |  | *4414* | | | |

Note: 1) Linear probability model with household fixed effects, controlling for time dummies and weekend/weekdays. 2) Confidence intervals based on cluster-robust standard errors grouped at the health facility level; *** p<0.01, ** p<0.05, * p<0.1

**Table F. Alternative model: household random-effects logistic model**

|  | A. Full sample | | | |  | B. Under-five children | | | |
| --- | --- | --- | --- | --- | --- | --- | --- | --- | --- |
| *Health Care Seeking Behavior* | Mean | OR | p-value | 95% CI |  | Mean | OR | p-value | 95% CI |
| Use the facility | 0.300 | 0.809 | 0.219 | [0.577, 1.134] |  | 0.304 | 0.828 | 0.319 | [0.571, 1.201] |
| Seek facility-based care | 0.393 | 0.976 | 0.886 | [0.702, 1.357] |  | 0.391 | 0.996 | 0.982 | [0.685, 1.447] |
| Go to public facilities | 0.275 | 0.889 | 0.532 | [0.616, 1.284] |  | 0.272 | 0.979 | 0.926 | [0.626, 1.530] |
| Go to high level public facilities | 0.092 | 0.561 | 0.208 | [0.228, 1.380] |  | 0.085 | 0.221*** | 0.007 | [0.073, 0.666] |
| Go to low level public facilities | 0.169 | 0.951 | 0.831 | [0.602, 1.504] |  | 0.177 | 1.346 | 0.244 | [0.817, 2.217] |
| Go to private facilities | 0.153 | 1.009 | 0.965 | [0.667, 1.529] |  | 0.155 | 0.79 | 0.450 | [0.428, 1.458] |
| Go to retail sectors | 0.396 | 1.238 | 0.169 | [0.914, 1.678] |  | 0.394 | 1.377 | 0.153 | [0.887, 2.137] |
| *Testing and Treatment* |  |  |  |  |  |  |  |  |  |
| Receive malaria test | 0.272 | 0.728** | 0.030 | [0.547, 0.970] |  | 0.290 | 0.73 | 0.166 | [0.468, 1.139] |
| Take medications | 0.757 | 1.343* | 0.058 | [0.990, 1.820] |  | 0.749 | 1.773*** | 0.002 | [1.226, 2.565] |
| Take any antimalarial drugs | 0.539 | 1.053 | 0.645 | [0.845, 1.314] |  | 0.587 | 1.531** | 0.013 | [1.095, 2.142] |
| Take antibiotics | 0.270 | 1.258* | 0.070 | [0.981, 1.612] |  | 0.277 | 1.589** | 0.014 | [1.098, 2.299] |
| Pay OOP for medications | 0.359 | 1.423** | 0.011 | [1.084, 1.866] |  | 0.373 | 1.502** | 0.039 | [1.020, 2.211] |
| Illness lasts more than a week | 0.209 | 1.065 | 0.621 | [0.829, 1.370] |  | 0.194 | 1.187 | 0.362 | [0.821, 1.715] |
| *N* | *11704* | | | |  | *4637* | | | |

Note: 1) Household random effects model controls for time dummies and weekend/weekdays. 2) Confidence intervals based on cluster-robust standard errors grouped at the health facility level; *** p<0.01, ** p<0.05, * p<0.1

**Table G. Alternative model: generalized estimating equation (GEE) model**

|  | A. Full sample | | | |  | B. Under-five children | | | |
| --- | --- | --- | --- | --- | --- | --- | --- | --- | --- |
| *Health Care Seeking Behavior* | Mean | OR | p-value | 95% CI |  | Mean | OR | p-value | 95% CI |
| Use the facility | 0.300 | 0.874 | 0.346 | [0.661, 1.156] |  | 0.304 | 0.794 | 0.140 | [0.584, 1.078] |
| Seek facility-based care | 0.393 | 1.048 | 0.712 | [0.817, 1.344] |  | 0.391 | 0.942 | 0.666 | [0.717, 1.236] |
| Go to public facilities | 0.275 | 0.950 | 0.750 | [0.695, 1.300] |  | 0.272 | 0.895 | 0.532 | [0.632, 1.267] |
| Go to high level public facilities | 0.092 | 0.890 | 0.533 | [0.616, 1.285] |  | 0.085 | 0.607** | 0.030 | [0.386, 0.953] |
| Go to low level public facilities | 0.169 | 0.898 | 0.590 | [0.606, 1.330] |  | 0.177 | 0.923 | 0.704 | [0.611, 1.395] |
| Go to private facilities | 0.153 | 1.052 | 0.597 | [0.873, 1.267] |  | 0.155 | 0.905 | 0.443 | [0.702, 1.167] |
| Go to retail sectors | 0.396 | 1.217 | 0.117 | [0.952, 1.556] |  | 0.394 | 1.291 | 0.157 | [0.906, 1.840] |
| *Testing and Treatment* |  |  |  |  |  |  |  |  |  |
| Receive malaria test | 0.272 | 0.832 | 0.176 | [0.637, 1.086] |  | 0.290 | 0.774 | 0.220 | [0.513, 1.166] |
| Take medications | 0.757 | 1.326** | 0.019 | [1.047, 1.678] |  | 0.749 | 1.544*** | 0.007 | [1.125, 2.118] |
| Take any antimalarial drugs | 0.539 | 1.115 | 0.179 | [0.951, 1.308] |  | 0.587 | 1.309** | 0.050 | [1.000, 1.714] |
| Take antibiotics | 0.270 | 1.206* | 0.085 | [0.975, 1.491] |  | 0.277 | 1.456** | 0.019 | [1.062, 1.995] |
| Pay OOP for medications | 0.359 | 1.297** | 0.011 | [1.062, 1.583] |  | 0.373 | 1.272 | 0.122 | [0.938, 1.727] |
| Illness lasts more than a week | 0.209 | 1.035 | 0.788 | [0.805, 1.330] |  | 0.194 | 1.165 | 0.443 | [0.789, 1.721] |
| *N* | *11704* | | | |  | *4637* | | | |

Note: 1) GEE with exchangeable correlation structure, controlling for time dummies and weekend/weekdays. 2) Confidence intervals based on cluster-robust standard errors grouped at the health facility level; *** p<0.01, ** p<0.05, * p<0.1

**Table H. Baseline characteristics of the included households and excluded households**

|  | **Excluded**  **Households**  **(n = 811)** | **Included**  **Households**  **(n = 1555)** | **Difference** | **p value** |
| --- | --- | --- | --- | --- |
| ***Household Baseline Characteristics*** |  |  |  |  |
| Mean number of household members | 6.00 | 6.81 | -0.820 | <0.001 |
| Number of households own at least one bicycle (%) | 454 (0.56) | 945 (0.61) | -0.048 | 0.029 |
| Number of households own at least one mobile phone (%) | 433 (0.53) | 939 (0.60) | -0.070 | 0.009 |
| Number of households own at least one radio (%) | 485 (0.60) | 949 (0.61) | -0.013 | 0.513 |
| Number of households have electricity (%) | 65 (0.08) | 131 (0.08) | -0.003 | 0.773 |
| Number of household own a mosquito net (%) | 649 (0.80) | 1267 (0.82) | -0.015 | 0.472 |
| Number of household heads read English (%) | 215 (0.27) | 465 (0.30) | -0.034 | 0.101 |
| Fraction of household members completing primary education | 0.24 | 0.24 | 0.002 | 0.894 |
| Fraction of household members sleeping under a mosquito net | 0.56 | 0.57 | -0.005 | 0.845 |
| ***Access to facilities*** |  |  |  |  |
| Number of public facilities within a 3km radius | 1.16 | 1.26 | -0.104 | 0.455 |
| Number of private facilities within a 3km radius | 1.14 | 1.55 | -0.412 | 0.010 |
| Number of retail sectors within a 3km radius | 5.81 | 7.38 | -1.574 | 0.001 |
| Distance to closest public facility (km) | 2.56 | 2.01 | 0.545 | 0.043 |
| Distance to closest private facility (km) | 4.25 | 3.27 | 0.983 | 0.089 |
| Distance to closest retail sector (km) | 0.96 | 0.85 | 0.111 | 0.505 |

Note: p-value adjusted for clustering at village level.
