# Supplementary material for: The impact of health worker absenteeism on patient health care seeking behavior, testing and treatment: A longitudinal analysis in Uganda
Source: PLoS One. 2021 Aug 20;16(8):e0256437. doi: 10.1371/journal.pone.0256437 (PMC8378719; doi:10.1371/journal.pone.0256437)
Supplement: S2 Appendix — (DOC) [file pone.0256437.s002.doc]

| 4.0 | **FOLLOW BACK SURVEY (ENGLISH)** | | | | |
| --- | --- | --- | --- | --- | --- |
| **READ TO RESPONDENT:** I am now going to ask you some questions about each person in your household that has been sick in the last 4 weeks. We will start with the first person listed on the household roster that you said was sick. I will ask you a number of questions about their recent illness. Please tell me about all illnesses of any kind (e.g., fever, diarrhea, cold/flu, respiratory infection) | | | | | |
|  | | What is the first name of the person who was sick? | [_______________________________] | | |
|  | | What is the surname of the person who was sick? | [_______________________________] | | |
|  | | What is the household ID number?  **Same as Household ID on cover page** | [__|__|__|__] | | |
|  | | What is the person’s ID number?  **ID number is found on roster from cover page** | [__|__] | | |
|  | | What was the health problem?  **If the respondent does not know the illness that they had, briefly describe the symptoms or problem.** |  | | |
|  | | When did the health problem start? | [__|__]-[__|__]-[__|__|__|__] DD-MM-YYYY  98 = Refused  99 = Don't know | | |
|  | | When did the health problem end? (Approximate Date) | [__|__]-[__|__]-[__|__|__|__] DD-MM-YYYY  97 = Symptoms still persist **SKIP to next Ill HH Member**  98 = Refused  99 = Don't know | | |
|  | | Did you/NAME have any of the following symptoms? | | | |
|  |  | Fever 1 = Yes 2 = No 9 = Don’t Know  Chills 1 = Yes 2 = No 9 = Don’t Know  Headache 1 = Yes 2 = No 9 = Don’t Know  Joint pain/Joint ache 1 = Yes 2 = No 9 = Don’t Know  Loss of Appetite 1 = Yes 2 = No 9 = Don’t Know | Diarrhea 1 = Yes 2 = No 9 = Don’t Know  Vomiting 1 = Yes 2 = No 9 = Don’t Know  Stomach Pains 1 = Yes 2 = No 9 = Don’t Know  Runny Nose/ 1 = Yes 2 = No 9 = Don’t Know  Congestion | | |
|  | | Can you show me on this ladder the level of pain you/NAME were feeling when you/NAME were sick? **Use Pain Scale Visual** | [__|__] Write in number respondent indicated on the ladder.  99 = Don’t Know | | |
|  | | Can you show me on this ladder the level of fever, or high temperature, you/NAME experienced when you/NAME were sick? **Use Fever Scale Visual** | [__|__] Write in number respondent indicated on the ladder.  99 = Don’t Know | | |
|  | | Can you show me on this ladder the level of fatigue, or how tired you/NAME were feeling when you/NAME were sick? **Use Fatigue Scale Visual** | [__|__] Write in number respondent indicated on the ladder.  99 = Don’t Know | | |
|  | | Can you show me on this ladder how bad the illness was overall? **Use Overall Illness Severity Scale Visual** | [__|__] Write in number respondent indicated on the ladder.  99 = Don’t Know | | |
|  | | **DO NOT READ OUT LOUD:**  Did the respondent answer “malaria” in Q 4.5? | | | 1 = Yes  2 = No |
|  | | **DO NOT READ OUT LOUD:**  Did the respondent answer “yes” to *any* of the symptoms asked in Q 4.8? | | | 1 = Yes  2 = No |
|  | | **DO NOT READ OUT LOUD:**  If the respondent answered “Yes” in Question 4.13, **or** Question 4.14, Circle 1 = Yes  If the respondent answered “No” in **both** questions 4.13 **and** 4.14, Circle 2 = No | | | 1 = Yes  2 = No **SKIP to 4.17** |
|  | | I'd like you to tell me how likely you think it was that your illness was malaria on this ladder. Where would you /NAME be on the scale? **Use Malaria Scale Visual** | [__|__] Write in number respondent indicated on the ladder.  99 = Don’t Know | | |
|  | | How did the illness interfere with your/NAME’s daily responsibilities? | 1 = Did not interfere at all  2 = Able to complete some of my chores  3 = Had to stay in bed - could not work at all  4 = Does not have daily responsibilities | | |
|  | | How many days were you/NAME sick? | [__|__] days  99 = Don't remember | | |
|  | | What did you/NAME do first? | | | |
|  |  | 1 = Took herbs or medicines from home  2 = Went to a Mobile Vendor **SKIP to 4.28**  3 = Went to pharmacy or drug shop **SKIP to 4.28**  4 = Went to see Community Health Worker **SKIP to 4.28**  5 = Went to Public Health Centre/Hospital **SKIP to 4.28**  6 = Went to Private hospital/clinic **SKIP to 4.28** | | 7 = Went to Traditional healer **SKIP to 4.28**  8 = Took herbs or medicine from neighbor  9 = Wait/Do nothing **SKIP to 4.21**  96 = Other *(specify)* [________________] **SKIP to 4.21**  98= Refused **END**  99 = Don’t know **END** | |
|  | | What herbs or medicines did you/NAME take?  **Write in all herbs and medicines the respondent took.** | | | |
|  |  | Medicine or Herb 1 [____________________________________] 98 = Refused  Medicine or Herb 2 [____________________________________] 99 = Don’t Know  Medicine or Herb 3 [____________________________________]  Medicine or Herb 4 [____________________________________]  Medicine or Herb 5 [____________________________________] | | | |
|  | | Did this resolve the illness? | | 1 = Yes **END**  2 = No | |
|  | | What did you/NAME do next? | | | |
|  |  | 1 = Took herbs or medicines from home  2 = Went to a Mobile Vendor **SKIP to 4.28**  3 = Went to pharmacy or drug shop **SKIP to 4.28**  4 = Went to see Community Health Worker **SKIP to 4.28**  5 = Went to Public Health Centre/Hospital **SKIP to 4.28**  6 = Went to Private hospital/clinic **SKIP to 4.28** | | 7 = Went to Traditional healer **SKIP to 4.28**  8 = Took herbs or medicine from neighbor **SKIP to 4.24**  9 = Wait/Do nothing **SKIP to 4.25**  96 = Other *(specify)* [________________] **SKIP to 4.25**  98= Refused **END**  99 = Don’t know **END** | |
|  | | How many days did you/NAME wait? | | 1 = 1 day  2 = 2 days  3 = 3 or more days  98 = Refused  99 = Don't know | |
|  | | What herbs or medicines did you/NAME take?  **Write in all herbs and medicines the respondent took.** | | | |
|  |  | Medicine or Herb 1 [____________________________________] 98 = Refused  Medicine or Herb 2 [____________________________________] 99 = Don’t Know  Medicine or Herb 3 [____________________________________]  Medicine or Herb 4 [____________________________________]  Medicine or Herb 5 [____________________________________] | | | |
|  | | Did this resolve the illness? | 1 = Yes **END**  2 = No | | |
|  | | What did you/NAME do next? | | | |
|  |  | 1 = Took more herbs or medicine from home **END**  2 = Went to a mobile vendor  3 = Went to pharmacy or drug shop  4 = Went to see Community Health Worker  5 = Went to Public Health Centre/Hospital  6 = Went to Private hospital/clinic | 7 = Went to Traditional healer  8 = Wait/Do Nothing **END**  96 = Other *(specify)* [__________________] **END**  98= Refused **END**  99=Don’t know **END** | | |
|  | | How many days did you/NAME wait? | [__|__] days  99 = Don't know | | |

| **First Place** | | | | | | | | | | | |
| --- | --- | --- | --- | --- | --- | --- | --- | --- | --- | --- | --- |
| 4.28 | | | What is the name of the place you went to? | | | | [___________________________] | | | | |
| 4.29 | | | Where is the location of this place? **Write in name of village.** | | | | [___________________________] | | | | |
| 4.30 | | | Did you stay overnight? | | | | 1 = Yes  2 = No | | | | |
| **Malaria Testing: Enumerator: Check for any indication of malaria**   - **If you circled “2 = No” in Q 4.15 (there is no indication of malaria), SKIP to 4.38** | | | | | | | | | | | |
| 4.31 | | Did you take a malaria test at this place? | | | | | 1 = Yes  2 = No **SKIP to 4.38** | | 99 = Don’t know **SKIP to 4.38** | | |
| **4.32 Type of Test**  Use RDT and Slide Samples | | | | **4.33 What was the cost of the test?** | | | **4.34 Test Requested or Offered** | | | **4.35 What was the result of the test?** | |
| 1 = RDT  2 = Microscopy  99 = Don't know | | | | [__|__|__|__] UGX  98 = Refused  99 = Don't know | | | 1 = Requested  2 = Offered  99 = Don’t know | | | 1 = Positive  2 = Negative  3 = Inconclusive/No Result  98 = Refused  99 = Don’t know | |
| 4.36 | | Do you have a copy of your test results? | | | | | 1 = Yes 99 = Don’t know **SKIP to 4.38**  2 = No **SKIP to 4.38** | | | | |
| 4.37 | | **DO NOT READ OUTLOUD**  Record the result written on the result sheet | | | | | 1 = Positive  2 = Negative  3 = Inconclusive/No Result  98 = Refused to show | | | | |
| 4.38 | | | Did you obtain medicines from this place? | | | | 1 = Yes 99 = Don’t know **SKIP to 4.39**  2 = No **SKIP to 4.39** | | | | |
|  | **Medication Name** | | | | **Medication Code**  Use codes below | | **Prescription**  Indicate if this drug was bought with a prescription or advice from a doctor or nurse | | | | **Cost of Medication**  If it was free, write ‘00000’  Don’t Know – write ‘99999’ |
| **1** | [______________________________] | | | | [__|__] | | 1 = Yes 2 = No 99 = Don’t know | | | | [__|__|__|__|__] UGX |
| **2** | [______________________________] | | | | [__|__] | | 1 = Yes 2 = No 99 = Don’t know | | | | [__|__|__|__|__] UGX |
| **3** | [______________________________] | | | | [__|__] | | 1 = Yes 2 = No 99 = Don’t know | | | | [__|__|__|__|__] UGX |
| **Total Cost of Medication:** Fill in this line ONLY if the respondent cannot separate medication costs. Fill in if she can remember the total only. | | | | | | | | | | | [__|__|__|__|__] UGX |
| **Medication Codes:**  1 = Antibiotic (e.g., Amoxil/amoxicillin, ciprofloxacin, Septin, erythromycin, norfloxacin)  2 = Antiworm/ Protozoa (e.g., Flagyl and mebendazole)  3 = Painkiller* (Brufen, Action, Panadol, Hedex, Painex, and Paracetamol)  4 = Cough/Cold Meds (e.g., Cold Cap, Flugon, and Tricoff) | | | | | | 5= Antimalarial (ACT) (e.g., Coartem, Lonart, Artesunate, Alu, Artemethur Lumafantrine, etc.)  6 = Antimalarial (Monotherapy) (e.g. Amodiaquine)  7 = Other Antimalarial (e.g., Fansidar, Quinine, SP, Chloroquine) 96 = Other *(specify)*  98 = Refused  99 = Don't know | | | | | |
| 4.39 | | | Did you go to a SECOND place? | | | | | 1 = Yes  2 = No **GOTO next Ill HH Member** | | | |

| **Second Place** | | | | | | | | | | | |
| --- | --- | --- | --- | --- | --- | --- | --- | --- | --- | --- | --- |
| 4.40 | | | What is the name of the place you went to? | | | | [___________________________] | | | | |
| 4.41 | | | Where is the location of this place? **Write in name of village.** | | | | [___________________________] | | | | |
| 4.42 | | | Did you stay overnight? | | | | 1 = Yes  2 = No | | | | |
| **Malaria Testing: Enumerator: Check for any indication of malaria**   - **If you circled “2 = No” in Q 4.15 (there is no indication of malaria), SKIP to 4.50** | | | | | | | | | | | |
| 4.43 | | Did you take a malaria test at this place? | | | | | 1 = Yes 99 = Don’t know **SKIP to 4.50**  2 = No **SKIP to 4.50** | | | | |
| **4.44 Type of Test**  Use RDT and Slide Samples | | | | **4.45 What was the cost of the test?** | | | **4.46 Test Requested or Offered** | | | **4.47 What was the result of the test?** | |
| 1 = RDT  2 = Microscopy  99 = Don't know | | | | [__|__|__|__] UGX  98 = Refused  99 = Don't know | | | 1 = Requested  2 = Offered  99 = Don’t know | | | 1 = Positive  2 = Negative  3 = Inconclusive/No Result  98 = Refused  99 = Don’t know | |
| 4.48 | | Do you have a copy of your test results? | | | | | 1 = Yes 99 = Don’t know **SKIP to 4.50**  2 = No **SKIP to 4.50** | | | | |
| 4.49 | | **DO NOT READ OUTLOUD**  Record the result written on the result sheet | | | | | 1 = Positive  2 = Negative  3 = Inconclusive/No Result  98 = Refused to Show | | | | |
| 4.50 | | Did you obtain medicines from this place? | | | | | 1 = Yes 99 = Don’t know **SKIP to 4.51**  2 = No **SKIP to 4.51** | | | | |
|  | **Medication Name** | | | | **Medication Code**  Use codes below | | | **Prescription**  Indicate if this drug was bought with a prescription or advice from a doctor or nurse | | | **Cost of Medication**  If it was free, write ‘00000’  Don’t Know – write ‘99999’ |
| **1** | [______________________________] | | | | [__|__] | | | 1 = Yes 2 = No 99 = Don’t know | | | [__|__|__|__|__] UGX |
| **2** | [______________________________] | | | | [__|__] | | | 1 = Yes 2 = No 99 = Don’t know | | | [__|__|__|__|__] UGX |
| **3** | [______________________________] | | | | [__|__] | | | 1 = Yes 2 = No 99 = Don’t know | | | [__|__|__|__|__] UGX |
| **Total Cost of Medication:** Fill in this line ONLY if the respondent cannot separate medication costs. Fill in if she can remember the total only. | | | | | | | | | | | [__|__|__|__|__] UGX |
| **Medication Codes:**  1 = Antibiotic (e.g., Amoxil/amoxicillin, ciprofloxacin, Septin, erythromycin, norfloxacin)  2 = Antiworm/ Protozoa (e.g., Flagyl and mebendazole)  3 = Painkiller* (Brufen, Action, Panadol, Hedex, Painex, and Paracetamol)  4 = Cough/Cold Meds (e.g., Cold Cap, Flugon, and Tricoff) | | | | | | 5= Antimalarial (ACT) (e.g., Coartem, Lonart, Artesunate, Alu, Artemethur Lumafantrine, etc.)  6 = Antimalarial (Monotherapy) (e.g. Amodiaquine)  7 = Other Antimalarial (e.g., Fansidar, Quinine, SP, Chloroquine) 96 = Other *(specify)*  98 = Refused  99 = Don't know | | | | | |
| 4.51 | | | Did you go to a THIRD place? | | | | | | 1 = Yes  2 = No **GOTO Next Ill HH Member** | | |

| **Third Place** | | | | | | | | | |
| --- | --- | --- | --- | --- | --- | --- | --- | --- | --- |
| 4.52 | | What is the name of the place you went to? | | | | [___________________________] | | | |
| 4.53 | | Where is the location of this place? **Write in name of village.** | | | | [___________________________] | | | |
| 4.54 | | Did you stay overnight? | | | | 1 = Yes  2 = No | | | |
| **Malaria Testing: Enumerator: Check for any indication of malaria**   - **If you circled “2 = No” in Q 4.15 (there is no indication of malaria), SKIP to 4.62** | | | | | | | | | |
| 4.55 | | Did you take a malaria test at this place? | | | | 1 = Yes 99 = Don’t know **SKIP to 4.62**  2 = No **SKIP to 4.62** | | | |
| **4.56 Type of Test**  Use RDT and Slide Samples | | | **4.57 What was the cost of the test?** | | | **4.58 Test Requested or Offered** | | **4.59 What was the result of the test?** | |
| 1 = RDT  2 = Microscopy  99 = Don't know | | | [__|__|__|__] UGX  98 = Refused  99 = Don't know | | | 1 = Requested  2 = Offered  99 = Don’t know | | 1 = Positive  2 = Negative  3 = Inconclusive/No Result  98 = Refused  99 = Don’t know | |
| 4.60 | | Do you have a copy of your test results? | | | | 1 = Yes 99 = Don’t know **SKIP to 4.62**  2 = No **SKIP to 4.62** | | | |
| 4.61 | | **DO NOT READ OUTLOUD**  Record the result written on the result sheet | | | | 1 = Positive  2 = Negative  3 = Inconclusive/No Result  98 = Refused to Show | | | |
| 4.62 | | Did you obtain medicines from this place? | | | | 1 = Yes 99 = Don’t know **SKIP to 4.62**  2 = No **GOTO next ill Household Member** | | | |
|  | **Medication Name** | | | **Medication Code**  Use codes below | | | **Prescription**  Indicate if this drug was bought with a prescription or advice from a doctor or nurse | | **Cost of Medication**  If it was free, write ‘00000’  Don’t Know – write ‘99999’ |
| **1** | [______________________________] | | | [__|__] | | | 1 = Yes 2 = No 99 = Don’t know | | [__|__|__|__|__] UGX |
| **2** | [______________________________] | | | [__|__] | | | 1 = Yes 2 = No 99 = Don’t know | | [__|__|__|__|__] UGX |
| **3** | [______________________________] | | | [__|__] | | | 1 = Yes 2 = No 99 = Don’t know | | [__|__|__|__|__] UGX |
| **Total Cost of Medication:** Fill in this line ONLY if the respondent cannot separate medication costs. Fill in if she can remember the total only. | | | | | | | | | [__|__|__|__|__] UGX |
| **Medication Codes:**  1 = Antibiotic (e.g., Amoxil/amoxicillin, ciprofloxacin, Septin, erythromycin, norfloxacin)  2 = Antiworm/ Protozoa (e.g., Flagyl and mebendazole)  3 = Painkiller* (Brufen, Action, Panadol, Hedex, Painex, and Paracetamol)  4 = Cough/Cold Meds (e.g., Cold Cap, Flugon, and Tricoff) | | | | | 5= Antimalarial (ACT) (e.g., Coartem, Lonart, Artesunate, Alu, Artemethur Lumafantrine, etc.)  6 = Antimalarial (Monotherapy) (e.g. Amodiaquine)  7 = Other Antimalarial (e.g., Fansidar, Quinine, SP, Chloroquine) 96 = Other *(specify)*  98 = Refused  99 = Don't know | | | | |
