# Supplementary material for: The impact of health worker absenteeism on patient health care seeking behavior, testing and treatment: A longitudinal analysis in Uganda
Source: PLoS One. 2021 Aug 20;16(8):e0256437. doi: 10.1371/journal.pone.0256437 (PMC8378719; doi:10.1371/journal.pone.0256437)
Supplement: S3 Appendix — (DOC) [file pone.0256437.s003.doc]

| **Instructions for filling out this form** | |
| --- | --- |
| **Name of Health centre/Clinic** | Clearly indicate the name of the health facility |
| **District** | Indicate the name of the district in which the health centre is found |
| **Sub county** | Write the name of the sub county in which the health facility is located |
| **Name of person reporting** | Clearly write your name |
| **Telephone number** | Clearly write your telephone contact |
| **Date** | Write the correct date ( for example 1/4/2011) |
| **Number of staff members present** | Write the total number of staff who are at work for the day |
| **ACTs available** | Please circle Yes if ACTs are in stock or No if ACTs are not in stock for the day you are reporting |
| **Malaria blood slides available** | Slides are the devices used to collect samples to test for malaria using the microscope circle Yes if they are available or No if they are not available. |
| **Reagents available** | Reagents are the all of the consumables (stains, oil and water) used for testing for malaria using the microscope circle Yes if they are available or No if they are not available |
| **Other anti-malarials available** | Please circle Yes if they are there or No if they are not available |
| **Name of other anti-malarials available** | Write clearly the name(s) of the anti-malarial (s) that is/are available or write Not applicable if you answered no in the availability category |
| **RDTs available** | The RDT is a rapid test device used for testing for malaria; it does not require a microscope to perform. Circle Yes if they are in stock or No if they are not in stock |

| **Facility Identification** | | | | | | | | | | | |
| --- | --- | --- | --- | --- | --- | --- | --- | --- | --- | --- | --- |
|  |  | | Facility ID: [__|__|__] | | | | | | | | |
|  |  | | Name of facility | | |  | | | | | |
|  |  | | Name of person completing form | | |  | | | | Tel. no | |
|  |  | | District | | |  | | | | Code [__|__] | |
|  |  | | Sub-County | | |  | | | | Code [__|__] | |
| **Daily report** | | | | | | | | | | | |
| **Date (dd-mm-yy)** | | **Number of staff members present** | | **Malaria blood slides available (circle)** | **Reagents available (circle)** | | **RDTs available (circle)** | **ACTs available**  **(circle)** | **Other anti-malarials available** | | **Name of other anti-malarials available** |
|  | |  | | Yes No | Yes No | | Yes No | Yes No | Yes No | |  |
|  | |  | | Yes No | Yes No | | Yes No | Yes No | Yes No | |  |
|  | |  | | Yes No | Yes No | | Yes No | Yes No | Yes No | |  |
|  | |  | | Yes No | Yes No | | Yes No | Yes No | Yes No | |  |
|  | |  | | Yes No | Yes No | | Yes No | Yes No | Yes No | |  |
|  | |  | | Yes No | Yes No | | Yes No | Yes No | Yes No | |  |
|  | |  | | Yes No | Yes No | | Yes No | Yes No | Yes No | |  |
|  | |  | | Yes No | Yes No | | Yes No | Yes No | Yes No | |  |
|  | |  | | Yes No | Yes No | | Yes No | Yes No | Yes No | |  |
|  | |  | | Yes No | Yes No | | Yes No | Yes No | Yes No | |  |
|  | |  | | Yes No | Yes No | | Yes No | Yes No | Yes No | |  |
|  | |  | | Yes No | Yes No | | Yes No | Yes No | Yes No | |  |
|  | |  | | Yes No | Yes No | | Yes No | Yes No | Yes No | |  |
|  | |  | | Yes No | Yes No | | Yes No | Yes No | Yes No | |  |
|  | |  | | Yes No | Yes No | | Yes No | Yes No | Yes No | |  |
|  | |  | | Yes No | Yes No | | Yes No | Yes No | Yes No | |  |
|  | |  | | Yes No | Yes No | | Yes No | Yes No | Yes No | |  |
|  | |  | | Yes No | Yes No | | Yes No | Yes No | Yes No | |  |
|  | |  | | Yes No | Yes No | | Yes No | Yes No | Yes No | |  |
|  | |  | | Yes No | Yes No | | Yes No | Yes No | Yes No | |  |
|  | |  | | Yes No | Yes No | | Yes No | Yes No | Yes No | |  |
|  | |  | | Yes No | Yes No | | Yes No | Yes No | Yes No | |  |
|  | |  | | Yes No | Yes No | | Yes No | Yes No | Yes No | |  |
|  | |  | | Yes No | Yes No | | Yes No | Yes No | Yes No | |  |
|  | |  | | Yes No | Yes No | | Yes No | Yes No | Yes No | |  |
|  | |  | | Yes No | Yes No | | Yes No | Yes No | Yes No | |  |
|  | |  | | Yes No | Yes No | | Yes No | Yes No | Yes No | |  |
|  | |  | | Yes No | Yes No | | Yes No | Yes No | Yes No | |  |
|  | |  | | Yes No | Yes No | | Yes No | Yes No | Yes No | |  |
|  | |  | | Yes No | Yes No | | Yes No | Yes No | Yes No | |  |
|  | |  | | Yes No | Yes No | | Yes No | Yes No | Yes No | |  |
